# Antimicrobial Properties of Fennel By-Product Extracts and Their Potential Applications in Meat Products

**DOI:** 10.3390/antibiotics13100932

**Published:** 2024-10-01

**Authors:** Marica Egidio, Loriana Casalino, Filomena De Biasio, Marika Di Paolo, Ricardo Gómez-García, Manuela Pintado, Alma Sardo, Raffaele Marrone

**Affiliations:** 1 Department of Veterinary Medicine and Animal Production, University of Naples Federico II, 80138 Naples, Italy; marica.egidio@libero.it (M.E.); alma.sardo@unina.it (A.S.); raffaele.marrone@unina.it (R.M.); 2 Department of Economic and Legal Sciences, Universitas Mercatorum, 00186 Rome, Italy; loriana.casalino@libero.it; 3 EVRA S.r.l. Società Benefit, 85044 Lauria, Italy; filomena.debiasio@evra-ingredients.com; 4 CBQF—Centro de Biotecnologia e Química Fina—Laboratório Associado, Escola Superior de Biotecnologia, Universidade Católica Portuguesa, Rua Diogo Botelho 1327, 4169-005 Porto, Portugal; rgarcia@ucp.pt (R.G.-G.); mpintado@ucp.pt (M.P.)

**Keywords:** minced meat products, additives, natural compounds, fennel extracts

## Abstract

**Background:** Beef burgers are perishable meat products, and to extend their shelf life, EU Regulation 1129/11 permits the use of certain additives. **Objectives:** However, given the concerns of health-conscious consumers and the potential toxicity of synthetic substances, this study aimed to explore the use of fennel waste extracts as natural preservatives. **Methods:** This study characterized the bioactive compounds (phenolic content), the antioxidant activity (ABTS^+^ and DPPH assay), and the antimicrobial properties (against *Salmonella enterica* serotype Enteritidis, *Escherichia coli*, *Staphylococcus aureus*, *Bacillus cereusi,* and *Pseudomonas aeruginosa*) of different fennel waste extracts (LF, liquid fraction; SF, solid fraction and PF, pellet fraction). Additionally, the potential use of the best fennel extract was evaluated for its impact on beef burger shelf life (up to 18 days at 4 ± 1 °C) in terms of microbiological profile, pH, and activity water (a_w_). **Results:** The PF extract, which was rich in flavones, hydroxybenzoic, and hydroxycinnamic acids, demonstrated the highest antioxidant and antimicrobial activities. Microbiological analyses on beef burgers with PF identified this extract as a potential antimicrobial substance. The a_w_ and pH values did not appear to be affected. **Conclusions:** In conclusion, fennel extracts could be proposed as natural compounds exploitable in beef burgers to preserve their quality and extend their shelf-life.

## 1. Introduction

Meat and meat products represent a significant source of energy and various nutrients in the human diet, such as high-value proteins containing essential amino acids, essential fatty acids, minerals (iron or zinc), and vitamins (B1, B2, B6, and B12) [1]. Their consumption is widespread globally, and they are commonly found in restaurants, fast food chains, and stores [2]. However, due to some physicochemical characteristics such as pH, water content, oxygen sensibility [3], and the production process, they are highly perishable products with a shorter shelf life compared to whole-muscle meat [4]. Microorganisms responsible for rapid microbiological spoilage lead to changes in flavor, odor, texture, color, and a reduction in shelf life [5]. In addition, microorganisms responsible for microbiological deterioration not only cause economic losses to the food industry, but some also pose health risks to consumers (*Salmonella* spp., *Staphylococcus aureus*, *Escherichia coli*, *Campylobacter jejuni*, *Listeria monocytogenes*, *Clostridium perfringes*, *Yersinia enterocolitica,* and *Aeromonas hydrophila*) [6]. Moreover, during storage, burgers also undergo natural chemical spoilage, characterized by lipid oxidation and protein degradation, resulting in nutrient loss and the development of unpleasant odors and flavors, further reducing their shelf life [7]. Therefore, given the economic importance of these meat products and high consumer demand, the primary concern of the food industry is to reduce and control microbial proliferation and chemical changes, thereby enhancing shelf life and overall safety [8]. Traditional meat preservation techniques include the use of synthetic chemical antioxidant and/or antimicrobial additives and preservatives, such as sulfur dioxide–sulphates, acetic acid, sorbates, and benzoates. These substances “prolong the shelf-life of foods by protecting them against deterioration caused by microorganisms and/or which protect against growth of pathogenic microorganisms.” [9]. In this regard, the addition of nitrates and nitrites to meat products offers several benefits, including enhanced quality characteristics and improved microbiological safety. These compounds, in fact, play a crucial role in developing the distinctive flavor, maintaining the red color stability, and protecting against lipid oxidation in cured meats [10]. Nitrites, in particular, exhibit significant bacteriostatic and bactericidal properties against various spoilage bacteria and foodborne pathogens commonly found in meat products and prevent the growth and toxin production of *Clostridium botulinum*. However, the International Agency for Research on Cancer (IARC) has recently determined that ingested nitrates or nitrites can be probable human carcinogens under conditions that promote endogenous nitrosation. Although the use of synthetic preservations offers numerous technological benefits and are thus of great importance to the meat industry, the safety of these chemical agents is questionable [11]. Concerns persist about the potential risks associated with their consumption, such as allergic reactions, gastrointestinal issues, carcinogenicity, asthma, and behavioral disorders like hyperactivity [12,13,14]. For this reason, the development and application of novel technologies involving natural compounds with antimicrobial and antioxidant properties have become a new strategy adopted by the meat industry to produce healthier products. These products are free from synthetic chemical additives or preservatives yet still offer appealing colors and flavors [15,16,17,18]. Numerous natural antimicrobial substances are already in use today, including bacteriophages and their lysins [19,20], bacteriocins [21], and plant extracts (including essential oils) [22,23]. In this regard, extracts from medicinal and aromatic plants, rich in bioactive compounds (e.g., phenolics, terpenoids, carotenoids) with potent activity against microbial growth and oxidative reactions, are increasingly being used as natural additives and quality enhancers in minced meat processing [24,25,26].

Fennel (*Foeniculum vulgare* Mill.) is one of these medicinal and aromatic plants, renowned for its anticancer, antidementia, antiplatelet, antihirsutism, hepatoprotective, and anti-hyperlipidemic properties due to its chemical composition [27,28]. Fennel extracts, particularly from fennel leaves, are important sources of vitamins (A, C, thiamine, riboflavin, and niacin), minerals (potassium, sodium, calcium, and manganese) [29], and n-3 fatty acids (primarily found in the leaves) [30]. Rich in bioactive compounds (phenolic acids, tocopherols, flavonoids, terpenoids, carotenoids) and essential oils [31,32,33], these extracts have significant potential as preservatives (antioxidative and antimicrobial) in the food processing sector [34,35,36]. Notably, Yanan Sun et al. [36], in a study focused on the meat industry, demonstrated the antimicrobial effectiveness of fennel extracts against aerobic mesophilic bacteria in ground pork. The study showed that fennel extracts could be used to improve the quality of ground pork and extend its shelf life.

Recently, new attention has shifted towards the search for sustainable solutions for recycling and valorizing food by-products for their reintegration into industrial supply chains [37]. However, despite the potential of by-products of fruits and vegetables, little attention has been given to studying and utilizing this waste and its potential. Therefore, this study aimed to explore the use of fennel waste extracts as natural preservatives. This study characterized the bioactive compounds, the antioxidant activity, and the antimicrobial properties (against *Salmonella enterica* serotype Enteritidis, *Escherichia coli*, *Staphylococcus aureus*, *Bacillus cereus,* and *Pseudomonas aeruginosa*) of different fennel waste extracts. Additionally, the potential use of the best fennel extract was evaluated for its impact on beef burger shelf life (up to 18 days at 4 ± 1 °C) in terms of microbiological profile, pH, and activity water (a_w_).

## 2. Results and Discussion

### 2.1. Total Phenolic and HPLC Analyses of the Phenolic Compounds in the Fennel Extracts

The results for the total phenolic content (TPC) and HPLC-DAD analysis are presented in Table 1. The data show that the fennel extract PF had the highest TPC, with 949 mg GAE/100 g DE, followed by the LF and SF fennel extracts, with 369.49 and 346.72 mg GAE/100 g DE, respectively. The total phenolic compounds measured based on the HPLC-DAD analysis were different and lower than those measured using the spectrophotometer based on the Folin–Ciocalteu assay (Table 1). These discrepancies can be attributed to the detection limits for certain phenolic compounds and to the fact that the Folin–Ciocalteu reagent can react with other molecules (such as sugars and proteins) present in the extracts, leading to measurement interference and an overestimation of total phenolic content. According to the HPLC-DAD analysis, four different chemical classes of polyphenols, including flavonols, flavones, and hydroxybenzoic and hydroxycinnamic acids, and seventeen individual phenolic compounds were identified in the three different fennel extracts (SF, LF, and PF), showing a high complexity and richness of such well-known bioactive compounds. Specifically, for each fennel fraction, at least nine of them (gallic acid, protocatechuic acid, catechin, dihydroxycaffeic acid, 4-hydroxybenzoic acid, caffeic acid, p-coumaric, ferulic acid, and rutin) were quantified using HPLC (Table 1). In this study, the most relevant phenolic compound was the protocatechuic acid hydroxybenzoic acid, whose highest concentration was found in the PF (126.89 mg/100 g DE). Moreover, 4-hydroxybenzoic acid and catechin (87.17 and 79.54 mg/100 g DE, respectively), which are bioactive polyphenols with antimicrobial, anti-inflammatory, and antioxidant properties attributed to their high free radical-scavenging capacity, were the most prominent compounds in the PF fennel extract. These higher PF values could be attributed to the presence of more lipophilic compounds, such as flavonoids (naringinin-7-glucoside, rutin, quercetin, and luteolin), which are associated with insoluble fiber and were separated during the milling and centrifugation process. Our data are consistent with previous research, such as the study by Roby et al. [38], which identified fifteen individual compounds, including quercetin, chlorogenic acid, and ferulic acid, and reported a lower total polyphenol content (TPC) in methanolic fennel extracts—340 mg GAE/100 g DE—compared to what was observed in this study. Similarly, Salami et al. [39] reported a high concentration of phenolic compounds (including chlorogenic acid, caffeic acid, p-coumaric acid, rutin, ferulic acid, 1,5-dicaffeoylquinic acid, quercetin, and apigenin) in fennel leaves and seeds, with TPC values of 200 mg GAE/100 g DE and 262 mg GAE/100 g DE, respectively.

### 2.2. Antioxidant Activity of the Fennel Extracts

The antioxidant activity of the free radical-scavenging fennel extracts is illustrated in Table 1. The results show that SF and PF had the highest antioxidant capacities compared to the LF extract. Specifically, SF exhibited strong scavenging effects on DPPH radicals, with values of 653.22 ± 14.66 μM TE/100 g DE, and on ABTS radicals, with values of 323.10 ± 25.11 mg AAE/100 g DE (dry extract). The PF extract, on the other hand, demonstrated the highest antioxidant activity on ABTS radicals, with values of 383.00 ± 18.07 mg AAE/100 g DE (dry extract), and on DPPH radicals, with values of 638.28 ± 46.70 μM TE/100 g DE. In contrast, the LF extract exhibited lower antioxidant activity, with scavenging effects on ABTS and DPPH radicals showing values of 328.85 ± 14.92 mg AAE/100 g DE (dry extract) and 143.64 ± 35.17 μM TE/100 g DE, respectively. These antioxidant activity results can be partly attributed to the high polyphenol content in the fennel extracts. Polyphenols are well-known for their potent ability to reduce oxidative compounds and scavenge free radicals, often surpassing the effectiveness of vitamins and carotenoids found in plants. Various studies have reported differing outcomes on this matter; while some authors have identified a correlation between phenolic content and antioxidant activity [40,41], others have found no such relationship, suggesting that other compounds may be responsible for the antioxidant effects [42]. Overall, the high antioxidant capacity observed in our study’s extracts highlights the importance of studying fennel by-products as a cost-effective and rich source of natural antioxidants.

### 2.3. Antimicrobial Screening of Fennel Fraction Extracts

**Figure 1:**
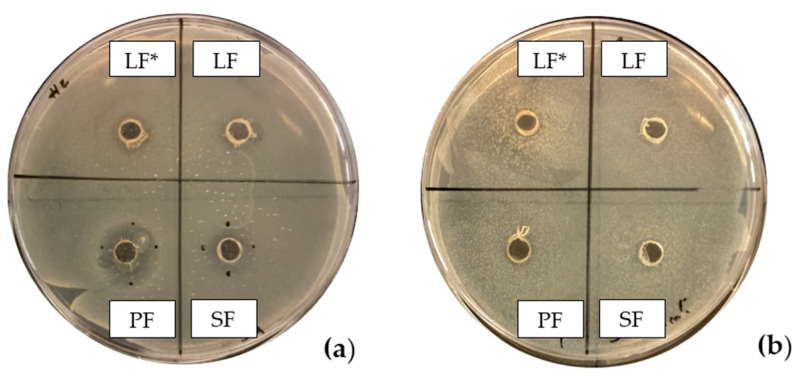
Inhibitory effects of fennel hydroalcoholic extracts (LF, liquid fraction used directly; LF*, liquid fraction at 40 °C; SF, solid fraction extract; PF, pellet fraction extract) against *Staphylococcus aureus* growth (**a**) and *Escherichia coli* (**b**).

The results illustrated in Figure 1 and Table 2 show the effects of the four fennel extracts (liquid fraction used directly, LF; liquid fraction at 40 °C, LF*; solid fraction, SF; and pellet fraction, PF) on the growth of *Staphylococcus aureus* and *Escherichia coli* in the first preliminary screening. Neither the directly used LF nor the concentrated LF* exhibited any inhibitory activity against the tested microorganisms. In fact, no inhibition zones were observed on the agar plates (Figure 1). Although a different concentration of the extracts was used in the screening test, making it difficult to compare antimicrobial activity, an inhibitory effect was observed for the SF and for PF extracts at concentrations of 110 mg DE/mL and 20 mg DE/mL, respectively, but only against *Staphylococcus aureus* (Figure 1a), with inhibition zones of 10 mm and 10.5 mm (Table 2). These data revealed less antimicrobial activity against *Staphylococcus aureus* compared to other studies. For example, Ghasemian et al., [43] reported that fennel essential oils exhibited an inhibition zone of 19 mm, while Barrahi et al., [44] found inhibition zones ranging from 10 to 20 mm. However, Lemiasheuski et al., [45], who also worked with essential oils extracted from various parts of the fennel plant (such as seeds, leaves, or aerial parts), reported inhibition zones of 5–6 mm, which were even smaller than those obtained in our study. It is important to note that the cited studies were focused on essential oils extracted directly from fennel, unlike our research, which examined whole extracts derived from fennel waste already used for other purposes. This difference could potentially explain the slight variations in our results compared to other studies in the field. In fact, it is well known that the antibacterial activity of a fennel extract is influenced by several different factors, including the fennel variety [33], the extraction method, and the part of the plant used for the extraction. For example, Ghafarizadeh et al., [46] observed that in the case of extracts obtained from fennel leaves, the ethanolic extract showed the highest inhibitory effect. Additionally, Rafieian et al., [47] demonstrated that extracts obtained from fennel flowers showed better antibacterial properties compared to the extracts obtained from other parts of the plant.

Moreover, in this first preliminary screening, none of the four extracts (LF, LF*, SF, PF) showed inhibitory effects against *Escherichia coli* (Figure 1b). Thus, the best result was obtained for the PF extract with the lowest concentration (20 mg DE/mL) and the higher inhibition (10.5 mm) only against *Staphylococcus aureus*, a Gram-positive bacterium. This result agrees with several previous studies [43,48] and has shown that fennel scrap extracts like fennel essential oils exhibit stronger antimicrobial activity against Gram-positive bacteria than Gram-negative bacteria. This difference in efficacy could be attributed to the distinct structural characteristics of their cell membranes. Gram-negative bacteria have a relatively thin peptidoglycan cell wall, which is further shielded by an outer membrane containing lipopolysaccharides. In contrast, Gram-positive bacteria lack this outer membrane but possess a much thicker peptidoglycan layer than that of Gram-negative bacteria [49,50].

After identifying the fennel extracts with antimicrobial properties (SF and PF) and their respective concentrations (50, 100, and 150 mg/mL for the SF; 10 and 20 mg DE/mL for PF), a second screening using a microplate-based assay was conducted. The data revealed that the fennel extracts (SF and PF) had inhibitory effects on the growth of all tested indicator strains (*Salmonella enterica* serotype Enteritidis, *Escherichia coli*, *Pseudomonas aeruginosa*, *Staphylococcus aureus,* and *Bacillus cereus*). Microbial growth, derived from absorbance data, decreased as the fennel extract concentration increased. As shown in Table 3, higher concentrations of the extracts resulted in greater growth inhibition, suggesting a proportional relationship between the amount of fennel extract used and its antimicrobial activity, as noted by Di Napoli et al., [51]. In summary, PF exhibited the best antimicrobial properties against all the tested microorganisms due to its effectiveness at lower concentrations (MIC = 2%) compared to the SF extract (MIC = 10%). In fact, an almost total inhibition (72–81%) of microbial growth was observed, particularly for the Gram-negative bacteria *Salmonella enterica* serovar Enteritidis, *Escherichia coli,* and *Pseudomonas aeruginosa*. Our data are consistent with other studies, which have confirmed the bactericidal activity of the fennel extract against these pathogens [51,52,53]. Moreover, in agreement with Ozcan et al., [54], the PF has shown its biocidal power against the Gram-positive *Bacillus cereus,* with an inhibition percentage (76%) similar to that observed for Gram-negative bacteria. Finally, an action, albeit minimal, was also observed on Gram-positive *Staphylococcus aureus*, with an inhibition of 28%. As the extracts were obtained from fresh fennel waste, this antimicrobial effectiveness could be attributed to the bioactive compounds present in the vegetable waste, such as hydroxybenzoic acids (gallic acid; 3,4 dihydroxybenzoic acid; protocatechuic acid; 4-hydroxybenzoic acid; *p*-coumaric and ferulic acid), hydroxycinnamic acids (dihydroxycaffeic acid and caffeic acid), flavonols (catechin), and flavonoids (rutin). Specifically, flavonoids are often reported to possess membrane-disrupting and inhibition activities of cell envelope synthesis, nucleic acid synthesis, electron transport chain, ATP synthesis, and biofilm formation [55], while the antimicrobial mechanisms of phenolic acids (hydroxybenzoic and hydroxycinnamic acids) are not yet fully understood. However, the leading theory suggests that these compounds destabilize microbial cell surfaces and cytoplasmic membranes, causing irreversible damage to the cell wall and various intracellular organelles. This process may lead to the coagulation of cellular components and the inhibition of intracellular enzymes. Additionally, once the cell wall is compromised, phenolic compounds may interact with intracellular components and DNA. The disruption of internal membranes can also release free radicals, which can further damage DNA and induce lipid oxidation [56].

Specifically, for *Salmonella enterica* serotype Enteritidis, an increase in the inhibitory index (%) was observed, directly proportional to the concentration of fennel extracts. The inhibition ranged from 44% to 82% for the SF extract and from 37% to 81% for the PF extract (Table 4). These findings are consistent with several studies investigating the antimicrobial effects of fennel plant extracts (notably essential oils) against *Salmonella enterica* serotype Enteritidis, demonstrating their inhibitory potentials on both the growth and virulence of this pathogen at different concentrations. For example, Pluta et al., [56] observed that fennel oils showed inhibitory effects at various concentrations, with the minimum inhibitory concentration (MIC) varying based on the specific strain and testing conditions. Moreover, Di Napoli et al., [51], in a study closely aligned with ours, revealed that fennel extracts, being rich in bioactive compounds, can significantly disrupt bacterial biofilms at low concentrations, showing strong antibacterial activity against various pathogens, including *Salmonella enterica*. Additionaly, Almuzaini [57] suggests that plant-derived compounds, including those found in fennel, such as phenolic compounds, polyphenols, flavonoid, terpenoids, and essential oils, can disrupt bacterial cell membranes, inhibit biofilm formation, and lead to bacterial death, particularly in strains like *Salmonella Typhimurium* and *Salmonella Enteritidis*.

Unlike the first preliminary screening, in the second one, both fennel extracts (SF and PF) showed antimicrobial effectiveness against *Escherichia coli*. An inhibitory index (%) ranging from 21% to 83% for the SF and from 0.12% to 72% for the PF was observed (Table 5). These data are in accordance with the study by Di Napoli et al. [51], where the fennel extract effectiveness was found to be dose-dependent, with higher concentrations leading to greater bacterial inhibition. According to Pluta et al., [56], this antimicrobial activity may be attributed to the bioactive components found in the extracts, which disrupt bacterial cell membranes and interfere with essential cellular processes. Notably, the mechanism involves disruption of the bacterium’s outer membrane, which, although more resistant due to its lipopolysaccharide layer, can still be permeated by the extract’s hydrophobic compounds under appropriate conditions [56]. Additionally, it is important to highlight that among all the Gram-negative bacteria analyzed in the present study, *Escherichia coli* was the most resistant to fennel extracts, as also observed Shabnam et al. [58]. However, unlike the study carried out by Manonmani and Khadir [59], which found that the ethanolic fennel extracts did not exhibit antibacterial effects against *Escherichia coli*, we found antimicrobial activity of the extracts even at a concentration of 20 mg DE/mL (MIC = 2%). The obtained MIC values are comparable to those reported by Gheorghita et al., (5–10%) [60].

Regarding the solid fraction, a similar pattern to that observed for *Escherichia coli* was noted for *Pseudomonas aeruginosa,* with an inhibitory index (%) ranging from 33% to 79% (Table 6). However, this Gram-negative bacterium proved to be more sensitive than *Escherichia coli* to the PF extract, with an inhibitory index (%) ranging from 19% to 75% (Table 5). This finding agrees with the study carried out by Daniela Gheorghita et al., [60] which reported the greater susceptibility of *Pseudomonas aeruginosa* compared to *Escherichia coli* regarding certain essential oils derived from various plants, including fennel, particularly in biofilm formation inhibition and growth reduction. In fact, the fennel’s ability to prevent biofilm formation, which is a key factor in *Pseudomonas aeruginosa’s* pathogenicity, disrupts its defense mechanism even at low concentrations, allowing for better control of bacterial growth [61]. Furthermore, generally, the minimum inhibitory concentrations (MIC) of essential oils for antibacterial activity may vary depending on the specific bacterial strain and the oil’s composition [61]. In our study, the MIC for PF (which is the best extract) was even lower (2 mg DE/mL) than that reported by Diao et al. [35] (>10 mg DE/mL), despite their research focusing on the antimicrobial activity of essential oils from fennel seeds (*Foeniculum vulgare* Mill.) and not on fennel scrap extracts.

Unlike the previous trends, the PF at a concentration of 10 mg DE/mL was unable to inhibit or reduce the proliferation of *Staphylococcus aureus*. In fact, for this microorganism, higher growth was observed compared to the untreated positive control (PC) (Table 3). At a concentration of 20 mg DE/mL, the PF showed slight efficacy, with an inhibitory index (%) of 28% (Table 7). For the SF (Table 3), the best result was observed at a concentration of 100 mg DE/mL (10%) with an inhibition of 43%, confirming the data obtained in the first preliminary screening (Figure 1 and Table 1). These results differ from those of Rafiejan et al., [47] and Barrahi et al., [44] who found that *Staphylococcus aureus* was among the bacteria most sensitive to fennel extracts. Conversely, Manonmani and Khadir reported that fennel ethanolic extracts showed no antibacterial activity against *Staphylococcus aureus*. In our study, however, the most effective ethanolic extract (PF) reduced microbial growth by 28%. According to Shahat et al., [62], these variations are likely due to differences in the concentrations used, extraction methods, and the specific parts of the plant analyzed. In fact, fennel extracts (notably essential oils) are effective in concentrations ranging from low to moderate, depending on the strain, the conditions, and the type of extract used.

For *Bacillus cereus*, the SF at a concentration of 100 mg DE/mL (10%) exhibited greater effectiveness than the SF at a concentration of 150 mg DE/mL (15%), with an inhibitory index of 72% (Table 8). For the PF, an increase in antibacterial activity directly proportional to the fennel extract concentrations was observed (Table 3), with an inhibitory index (%) ranging from 50% to 76% (Table 8). These findings are in accordance with Lemiasheuski et al., [45], who observed an inhibitory effect of approximately 50% on *Bacillus cereus* growth. The variation in antimicrobial efficacy may be attributed to differences in the type of extract used (essential oil versus ethanolic extract) and the concentration applied.

Overall, the data show that the maximum antibacterial activity was detected at a concentration of 20 mg DE/mL (2%) for the PF and 150 mg DE/mL (15%) for the SF, except for the Gram-positive bacteria *Bacillus cereus* and *Staphylococcus aureus*, where the best results were obtained at a concentration of 100 mg DE/mL (10%). Additionally, it is important to note that, unlike in the first preliminary screening, both fennel extracts demonstrated antibacterial effectiveness against *Escherichia coli* in this case, successfully reducing its proliferation.

### 2.4. Effects of Selected Fennel Extract on Beef Burgers

The preliminary in vitro screenings showed that the fennel leave extract with the best antimicrobial properties was the hydroalcoholic extract obtained from the semi-solid pellet. Based on these initial results, a shelf-life test was conducted in an Italian meat cutting and processing company to confirm its antimicrobial activity on beef burgers packaged in a skin pack for 18 days.

#### Microbiological Analysis

The results of the microbiological analysis of treated (TRT) and untreated (CTR) beef burgers are shown in Table 9. Overall, the data showed that all the analyzed parameters of the TRT beef burgers were not only recorded below the limits (m and M) set by the EC Regulation 1441/07 [63] but also exhibited lower values compared to those of CTR group (Table 9). However, no significant differences were found between CTR and TRT beef burgers (<1.0 Log (CFU/g)), likely because these products originated from the production facility and were prepared for commercial distribution, resulting in low contamination levels.

The TAB 30 °C of the TRT group exhibited an increasing trend during the storage until reaching a concentration equal to 5.32 Log (CFU/g) at 15 and 18 d. Despite this, it always remained lower than the TAB 30 °C registered for the CTR group (5.52 Log (CFU/g) at 18 d) and lower than 5.69 Log (CFU/g), which is the m limit set by EC Regulation 1441/07.

From 0 to 8 days, yeasts and molds showed a constant trend, with a concentration always below 1.0 Log (CFU/g). An increase in microbial proliferation was observed only at the end of the storage (from 15 to 18 d), but with concentrations (1.56 Log (CFU/g)) always lower than those obtained for the CTR group, which instead exhibited an increasing trend starting from day 8 (with 1.1 Log (CFU/g) at 8 d until 1.63 Log (CFU/g) at 18 d).

The other microbial determinations (coliforms, Beta-glucuronidase-positive *Escherichia coli*, coagulase-positive staphylococci, and *Listeria monocytogenes*) had a constant trend over time in both groups (CTR and TRT), always remaining below 1.0 Log (CFU/g) (respecting the m limits set by the EC Regulation 1441/07 [63] of 2.69 Log (CFU/g) for Beta-glucuronidase-positive *Escherichia coli* and of 2.0 Log (CFU/g) for *Listeria monocytogenes*). *Salmonella* spp. was not found at any time during the storage period. Similar findings were also reported by others [51,64,65] who evaluated the antimicrobial activity of extracts obtained from different parts of the fennel plant on meat products. Minor differences compared to other studies may be attributed to our use of extracts from fennel waste and their use of extracts or essential oils obtained directly from the plant.

With regard to the chemical analyses, pH and activity water (a_w_) are essential quality indicators for meat and meat products, as they impact the microbiological safety, water-holding capacity, and tenderness of the final products [26,66].

Throughout the storage period, the water activity (a_w_) values for the TRT group, ranging in intervals from 0.975 to 0.981, increased and dropped (from 0.979 at day 0 to 0.981 at day 8, 0.975 at day 12, 0.976 at day 15, and 0.975 at day 18) in narrow intervals, showing a fluctuating trend (Table 9). In comparison, the water activity (a_w_) values for the CTR group, ranging in intervals from 0.974 to 0.980, exhibited a gradual increase until day 12 before declining by day 18 (from 0.979 at day 0 to 0.980 at days 8 and 12, 0.975 at day 15, 0.974 at day 18).

Regarding pH values, both groups showed a decreasing trend over time. According to Huang et al., [67] this decline could be attributed to the activity of muscle and microbial enzymes, as well as the formation of organic acids. However, the TRT group showed a more pronounced pH reduction compared to the CTR group, except on the last two days (day 15 and day 18). Notably, on day 15, both groups had identical pH values (5.45), while on day 18, the CTR group had a slightly lower pH value (5.37) compared to the TRT group (5.40). This greater decrease in pH values observed in the TRT group may be due to a reduced rate of ammonia production, possibly influenced by the terpenoids present in natural extracts [67].

## 3. Materials and Methods

### 3.1. Reagents and Standards

Anhydrous sodium carbonate (Na_2_CO_3_) and Folin–Ciocalteu’s reagent were purchase from Merck (Algés, Portugal). Standards of gallic acid, caffeic acid, chlorogenic acid, p-coumaric acid, dihydroxicaffeic acid, ferulic acid, myricetin, quercetin, resveratrol, and rutin were acquired from Sigma-Aldrich (St. Louis, MA, USA), whereas catechin, (−epicatechin, epicatechin gallate, epigallocatechin gallate, epicatechin-3-O-gallate, hydroxytyrosol, kaempferol, and luteolin-7-glycosidewere purchased from Extrasyn these (Lyon, France). Formic acid and methanol were purchased from Fischer Scientific (Oeiras, Portugal).

### 3.2. Preparation of Vegetable Extracts

The vegetable extracts used in this study were provided by the Benefit Company Evra S.r.l (Lauria, PZ, Italy) and obtained according to the Patent No. 102021000007460. Briefly, fresh fennel (*Foeniculum vulgare* Mill.) scraps were centrifuged at 7000 rpm for 15 min at room temperature to obtain a liquid fraction and a solid fraction. The solid fraction was dried in a vacuum oven type M40-VT (MPM Instruments s.r.l.—Bernareggio, Italy) at 40 °C for 48 h and then ground into a fine powder using a grinder model WSG30E (Waring Commercial—Torrington, CT, USA). The liquid fraction was further centrifuged to obtain a semi-solid pellet and a liquid supernatant (LF). Subsequently, the powdered solid fraction and the semi-solid pellet were extracted with ethanol (30–70%) in a raw material to solvent ratio of 1:5 (g/mL) using a magnetic stirrer for 45 min at temperature ranging from 40 to 70 °C, resulting in extracts of the fennel solid (SF) and pellet fraction (PF). The obtained extracts were filtered and dried. For the experimental tests, the dried extracts (SF and PF) were reconstituted in distilled water to obtain hydroalcoholic fennel extracts at the required concentrations.

### 3.3. Total Phenolic Content (TPC)

The total phenolic content of the three extracts (SF, LF, and PF) was determined using the Folin–Ciocalteu method with some modifications, as reported by Gómez-García et al., [68]. In a 96-well plate, 20 μL aliquots of each sample were mixed with 80 μL of Folin–Ciocalteu reagent previously diluted 1:10 (*v*/*v*) in water and 100 μL of 7.5% (*w*/*v*) sodium carbonate. After incubating the mixture in the dark at room temperature for 1h, the absorbance was measured at 750 nm using a microplate reader (Synergy H1, Winooski, VT, USA). Gallic acid (Sigma-Aldrich, St. Louis, MA, USA) was used as the standard, and the results were expressed as mg gallic acid equivalents (GAE) per 100 g of dry extract (DE). Gallic acid was used as a calibration curve standard (0.05–0.50 mg/mL). All measurements were performed in triplicate for each experiment.

### 3.4. Analysis of Phenolic Compounds Using HPLC

The polyphenolic profile of fennel extracts (SF, LF, and PF) was obtained using high-performance liquid chromatography coupled to a diode-array detector (HPLC-DAD) according to the method described by Campos et al., [69] with some modifications. The samples were injected into a Waters Series e2695 Separation Module System (Mildford, MA, USA) interfaced with a UV/Vis photodiode array detector (PDA 190–600 nm). Separation was performed using a reverse-phase column (COSMOSIL 5C1 8-AR-II Packed Column—4.6 mm I.D. × 250 mm: Dartford, UK). The chromatographic separation of phenolic compounds was carried out with mobile phase A—water/methanol/formic acid (92.5:5:2.5, *v*/*v*/*v*) and mobile phase B—methanol/water/formic acid (92.5:5:2.5, *v*/*v*/*v*) under the following conditions: 50 μL of sample was injected at a continuous flow rate of 0.5 mL/min, with gradient elution starting at 100% mobile phase A for 50 min. From 50 to 55 min, the gradient was adjusted to 45% A and 55% B, then mobile phase A was returned to 100% for 4 min (until 59 min). Data acquisition and analysis were carried out using Empower 3 software. Measurements were taken at wavelengths ranging from 200 to 600 nm. Phenolic compounds were identified and quantified using an external calibration curve of each specific phenolic compound at concentrations ranging from 0.008 to 0.125 mg/mL by comparing retention times, UV absorption spectra, and peak areas with pure standards. All measurements were performed in triplicate for each experiment, and the results were expressed as mg of phenolic compounds per 100 g of dry extract (DE).

### 3.5. Antioxidant Activity of Fennel Fraction Extracts

The DPPH-radical scavenging activity of the fennel extracts (LF, SF, and PF) was determined according to the method described by Gómez-García et al., [68]. Briefly, 1.75 mL of DPPH solution (60 μM) was mixed with 250 μL of each extract, and the mixture was incubated at room temperature for 30 min in the dark. Then, the absorbance was measured at 515 nm using a UV spectrophotometer (Shimadzu UV-2401PC) (Kyoto, Japan). Trolox was used as a standard for the preparation of a calibration curve (0.005–0.08 mg/mL), and the results were expressed in μM of Trolox equivalents (TE) per 100 g of dry extract (DE). All measurements were performed in triplicate for each experiment.

The ABTS^+^ radical method was performed following the method described by Gómez-García et al., [68] mixing 10 μL of the sample extract with 1 mL of ABTS radical solution. After 6 min in the dark, the absorbance was recorded at 734 nm using a UV spectrophotometer. The stock solution was prepared by combining ABTS+ (7 mM) with potassium persulfate (2.5 mM) in ultra-pure water, and the mixture was stirred at room temperature for 16 h. The ABTS+ radical solution was subsequently diluted with water to achieve an absorbance of 0.700 ± 0.020 at 734 nm. The results were expressed in mg ascorbic acid (EAA) per 100 g of dry extract (DE) equivalents. The standard curve was made with l-ascorbic acid (0.05–0.5 mg/mL). All measurements were performed in triplicate for each experiment.

The antioxidant activity was calculated as follows:

Antioxidant activity = (A_blank_ − A_sample_/A_blank_) × 100

### 3.6. Antimicrobial Screening of Fennel Fraction Extracts

The antimicrobial properties of fennel leaves extract were evaluated in vitro against pathogenic and spoilage microorganisms commonly found in the meat industry. Two preliminary screenings (agar-well diffusion assay and microplate-based assay) were carried out to test the antibacterial activity of the fennel extracts (LF, PF, and SF) against the following microorganisms: *Salmonella enterica* serotype Enteritidis ATCC 13076, *Escherichia coli* ATCC 25922, *Staphylococcus aureus* ATCC 25923, *Bacillus cereus* NCTC 2599, and *Pseudomonas aeruginosa* from the CBQF-ESB collection. Moreover, a fourth extract (LF*), obtained by concentrating the LF using a rotary evaporator under a vacuum at a temperature below 40 °C, was tested using the agar-well diffusion method.

#### 3.6.1. Inoculum Preparation

All microbial strains used as indicators, stored in a freezer at −80 °C, were reactivated on a solid non-selective nutrient medium (Nutrient Agar, provided by Biolife, Milan, Italy) to assess their purity and vitality. According to ISO 4833-1:2013 [70], sterilized Petri dishes (9 cm in diameter) containing Plate Count Agar (PCA) were inoculated with the microbial indicator strains and incubated at 37 °C for 24 h. Bacterial cells were then picked from the colonies on the plates and suspended in phosphate buffer (pH 7.4) via agitation, aiming to achieve a final concentration of 1.5 × 10^8^ CFU/mL (0.5 McFarland standard), measured using a densitometer (Sensititre—Nephelometer, Thermo Fisher, Roskilde, Denmark).

#### 3.6.2. Agar-Well Diffusion Assay

A preliminary screening was performed using the agar-well diffusion method to test the antimicrobial properties of four fennel hydroalcoholic extracts (liquid fraction used directly, LF; liquid fraction concentrated, LF*; solid fraction, SF; and pellet fraction, PF) at different concentrations (38.7 mg DE/mL for LF, 160 mg DE/mL for LF*, 110 mg DE/mL for SF, and 20 mg DE/mL for PF) against the Gram-positive bacterium *Staphylococcus aureus* and Gram-negative *Escherichia coli*. The aim of this laboratory assay was to identify the most effective extracts and concentrations. Briefly, a standardized inoculum suspension of each bacterial strain, with a final concentration of 1.5 × 10^8^ CFU/mL, was swabbed onto PCA agar plates. Subsequently, four-millimeter wells were then created and inoculated with 50 μL of each hydroalcoholic extract. The plates were incubated for 24 h at 37 °C. The effectiveness of these extracts was evaluated by measuring the diameter of the inhibition zones.

#### 3.6.3. Microplate-Based Assay

After identifying the two most effective fennel extracts (SF and PF) and their respective concentrations (50, 100, and 150 mg/mL for the SF; 10 and 20 mg of dry extract DE/mL for PF), a second screening using a microplate-based assay was conducted to evaluate their antibacterial activity against pathogenic and spoilage microorganisms commonly found in the meat industry: *Salmonella enterica* serotype Enteritidis ATCC 13076, *Escherichia coli* ATCC 25922, *Pseudomonas aeruginosa*, *Staphylococcus aureus* ATCC 25923, and *Bacillus cereus* NCTC 2599. Briefly, for each fennel extract, various solutions at different concentrations were prepared using distilled water as follows: SF at concentrations of 50 mg DE/mL = 5%, 100 mg DE/mL = 10%, and 150 mg DE/mL = 15%, and PF at concentrations of 10 mg DE/mL = 1% and 20 mg DE/mL = 2%; the best inhibitory concentration being considered as the minimum inhibitory concentration (MIC). The MIC value corresponded to the lowest extract concentration that inhibited visible bacterial growth. The sterilized samples were added to the wells of sterile polystyrene 96-well microtiter plates (Sarstedt, Wexford, Ireland). Mueller–Hinton broth with the inoculum (without any extract solution) was used as the positive control, while Mueller–Hinton broth without the inoculum was used as the negative control (blank). The plates were incubated at 37 °C for 24 h. Absorbance was measured at 660 nm at 1 h intervals using a Thermo Scientific™ Multiskan™ GO Microplate Spectrophotometer (Roskilde, Denmark). The laboratory test was conducted in triplicate. According to Sahar Roshanak et al., [71] and María-Leonor Pla et al., [72], the increase in turbidity is a sign of microorganism growth. Thus, based on these studies and in accordance with Ju-Sung Kim et al., [66], we calculated the percent inhibition (%) of each fennel extract at the different concentrations (SF at concentrations of 50 mg DE/mL = 5%, 100 mg DE/mL = 10%, and 150 mg DE/mL = 15% and PF at concentrations of 10 mg DE/mL = 1% and 20 mg DE/mL = 2%) using the measured absorbance as follows:

Inhibition rate (%) = (1 − (*Abs_sample_ − Abs_blank_*)/*Abs_control_*)) × 100

where *Abs_sample_* is the absorbance of the experimental sample, *Abs_blank_* is the absorbance of the blank, and *Abs_control_* is the absorbance of the positive control.

### 3.7. Evaluation of Effects of Selected Fennel Extracts on Beef Burgers

The preliminary in vitro screenings showed that the fennel extract with the best antimicrobial properties was the hydroalcoholic extract obtained from the semi-solid pellet represented by the pellet fraction (PF). Consequently, to evaluate the effects of this extract on beef burgers, microbiological and physicochemical analyses were conducted. The results of the treated beef burger (TRT) analyses were compared to those of an untreated beef burger (control, CTR) commonly produced by the meat industry involved in this study.

#### 3.7.1. Beef Burger Processing and Analyses

The tested beef burgers were taken from a batch produced by the meat product company of interest. The following ingredients were used for the preparation of the dough: minced beef (84%), water (8.5%), salt (1.5%), and a powdered mixture of aromas, vegetable fibers, plant extracts, and potato starch (6%). In the TRT group, 2% (20 mg DE/mL) of the best fennel extract (PF) was added to the beef burgers and thoroughly mixed. The percentage refers to final product concentration. Beef burgers with no additions were used as controls (CTR). The mixture was then manually blended to obtain a homogeneous mass. The samples were divided into treated (TRT) and untreated (control, CTR) groups. All burgers were formed using a conventional burger maker, packaged in skin packs, and stored for 18 days (shelf life determined by the company based on preliminary studies) in a relative vacuum at a refrigerator temperature of 4 ± 1 °C (in a refrigerator with digital control of air temperature and relative humidity). When opening each package, the product’s acceptability was evaluated based on the color and smell to determine its suitability for analysis. Analyses were performed at 0, 5, 8, 12, 15, and 18 days from production. Sampling times were chosen based on preliminary shelf-life studies (data not published). At the laboratory, during when each package was opened from 0 to 18 days, microbiological and chemical analyses were carried out in triplicate.

#### 3.7.2. Microbiological and Chemical Analysis

In the microbiological laboratory, the following meat hygiene indicator microorganisms were determined: total aerobic plate count at 30 °C (TAB 30 °C), total coliforms, beta-glucuronidase-positive *Escherichia coli*, coagulase-positive staphylococci, yeasts, and molds. Briefly, 10 g of each sample was added to 90 mL (1:10, *w*/*v*) of sterilized peptone water (PW, CM0009, OXOID, Basingstoke, UK) in a sterile stomacher bag and homogenized at 230 rpm for three minutes in a peristaltic homogenizer (BagMixer^®^400 P, Interscience, Saint Nom, France). Subsequently, ten-fold serial dilutions were prepared from each homogenate to isolate and enumerate the following: the total aerobic plate count at 30 °C on plate count agar (PCA; CM0325, Oxoid, Madrid, Spain) incubated at 30 °C for 48–72 h according to ISO 4833-1:2013 [70]; the total coliforms on violet–red bile lactose agar (VRBL, Oxoid, Madrid, Spain) incubated at 37 °C for 48 h in agreement with ISO 4831:2006 [73]; the β-glucuronidase-positive *Escherichia coli* on tryptone bile x-glucuronide agar (TBX, CM0945, Oxoid, Basingstoke, Hampshire, UK) incubated at 44 °C for 24–48 h in agreement with ISO 16649-2:2001 [74]; the coagulase-positive staphylococci on Baird–Parker agar (Oxoid, Madrid, Spain) at 37 °C for 24–48 h in agreement with ISO 6888-1:1999 [75]; and yeasts and molds on dichloran rose–Bengal chloramphenicol agar (DRBC, Oxoid, Madrid, Spain) incubated at 25 °C for 120/168 h according to ISO 21527-2:2008 [76].

In addition, the potential presence of two pathogens most commonly associated with meat products was also evaluated: *Listeria monocytogenes*, according to ISO 11290-1:2017 [77], and *Salmonella* spp., according to ISO 6579-1:2017 [78]. For this, 25 g of each meat sample was homogenized in 225 mL (1:10 *w*/*v*) of buffer peptone water (BPW, CM0509, Oxoid, Basingstoke, UK) and incubated for 24 h at 37 °C for the detection of *Salmonella* spp. and in 225 mL of half Fraser broth (HF, CM1053, Oxoid, Basingstoke, Hampshire, UK), incubated for 24 h at 30 °C for the detection of *Listeria monocytogenes.*

During the storage period, water activity (a_w_) and pH were evaluated using Aqualab 4 TE—Decagon Devices (METER Group, Inc., Pullman, WA, USA) and a pH-meter (Crison-Micro TT 2022, Crison Instruments, Barcelona, Spain) equipped with a glass insertion electrode, respectively. The pH values were measured by inserting the glass electrode 2 cm deep into the beef burgers. Each measurement was performed in triplicate (*n* = 3), and the mean value was recorded as the result. Moreover, the overall acceptability was assessed based on visual and odor evaluation of the samples.

### 3.8. Statistical Analysis

Statistical analyses were performed using SAS software version 6. 3rd ed. One-way ANOVAs were performed for the results of the fennel fraction extracts by comparing the inhibition halos of each extract against the indicator microorganisms tested. For the analysis of total phenols, individual phenolic content, and DPPH and ABTS activity, Student’s t-test was applied. Physicochemical and microbiological data of beef burger analyses were statistically analyzed using generalized linear mixed models (GLMMs), including the fixed effects of treatment (CTR and TRT) and time (T0, T1, T2, T3, T4, and T5). Each measurement was performed in triplicate (n = 3), taking the mean value as the result. The means were compared using the Tukey test (*p* < 0.05; *p* < 0.01). All data were presented as the mean (M) ± standard error (SE).

## 4. Conclusions

Overall, as observed in other studies [64], the preliminary results from the present work suggest that fennel scraps and their extracts have significant potential as natural antioxidant and antimicrobial agents in minced meat products such as beef burgers.

The growing interest in optimizing the use of food waste and by-products from agriculture and other food sectors presents substantial opportunities for recovering high-value compounds such as bioactive compounds. The fact that fennel extracts obtained from these by-products were shown to have very high antioxidant activity and inhibitory potentials against microorganisms commonly found in meat products opens new possibilities for using vegetable waste in the meat industry. These extracts could serve as natural antimicrobial agents that are healthier and more sustainable than the chemical additives currently used in industrial markets. In fact, they have the potential to extend the shelf life of minced meat products, enhance food safety, and transform waste into valuable resources, aligning with the principles of the circular economy. This approach could reduce food waste while generating novel, safe, and natural value-added products.

However, to fully realize this potential, given the relatively limited antimicrobial effects of fennel extracts (notably the pellet fraction) observed in the beef burger experiments of this study, optimization of the extraction process and incorporation methods in minced meat is necessary to improve their antimicrobial efficacy. Further studies, such as challenge tests, are required to support these preliminary findings and will be the focus of future research.

## 5. Patents

Patent no. 102021000007460 was filed on 26 March 2021.

## Figures and Tables

**Table 1:** HPLC-DAD analysis of individual phenolic compounds and antioxidant activity of the hydroalcoholic extracts from the fennel by-product fractions.

	Compound	Chemical Class	RT	Concentration (mg/100 g DE)		
				SF	LF	PF
1	Gallic acid	Hydroxybenzoic acid	14.07	22.45 ± 3.66	30.63 ± 1.58	30.05 ± 1.14
2	3,4 dihydroxybenzoic acid	Hydroxybenzoic acid	17.49	42.88 ± 10.90	24.17 ± 1.24	nd
3	Protocatechuic acid	Hydroxybenzoic acid	17.92	83.86 ± 36.54	32.11 ± 1.92	126.89 ± 7.56
4	Catechin	Flavonol	23.53	10.38 ± 3.62	27.34 ± 1.57	79.54 ± 4.58
5	Neochlorogenic acid	Hydroxycinnamic acids	25.57	ud	ud	ud
6	Chlorogenic acid	Hydroxycinnamic acids	28.00	ud	ud	ud
7	Dihydroxycaffeic acid	Hydroxycinnamic acids	29.29	45.98 ± 6.76	27.43 ± 1.39	79.80 ± 4.05
8	4-Hydroxybenzoic acid	Hydroxybenzoic acid	34.03	nd	90.90 ± 5.15	87.17 ± 4.58
9	Caffeic acid	Hydroxycinnamic acids	37.19	40.16 ± 0.30	9.53 ± 0.60	33.18 ± 2.05
10	*p*-Coumaric	Hydroxybenzoic acid	43.80	38.2 ± 6.41	36.35 ± 2.27	56.71 ± 3.37
11	Ferulic acid	Hydroxybenzoic acid	48.63	ud	22.81 ± 1.12	26.13 ± 0.90
12	Myricetin	Flavonoid	50.53	ud	nd	ud
13	Naringinin-7-glucoside	Flavones	52.45	ud	ud	ud
14	Naringinin	Flavones	53.45	ud	ud	ud
15	Rutin	Flavonoid	54.66	15.28 ± 12.51	2.09 ± 0.15	6.08 ± 0.45
16	Quercetin	Flavonol	55.13	ud	ud	ud
17	Luteolin	Flovones	56.46	ud	ud	ud
Total by HPLC				298.69 ± 24.16	303.37 ± 17.00	525.25 ± 29.05
TPC (mg GAE/100 g DE)				346.72 ± 17.67	369.49 ± 12.76	949.29 ± 114.37
Antioxidant activity						
ABTS (mg AAE/100 g DE)				323.10 ± 25.11	328.85 ± 14.92	383.00 ± 18.07
DPPH (μM TE/100 g DE)				653.22 ± 14.66	143.64 ± 35.17	638.28 ± 46.70

RT: retention time; LF, liquid fraction used directly; SF, solid fraction extract; PF, pellet fraction extract; TPC, total phenolic content; GAE, gallic acid equivalents; DE, dry extract; AAE, ascorbic acid equivalents; TE, trolox equivalents; nd: not detected; ud; under detection limit. All determinations were carried out in triplicate, and results are shown as mean value ± standard deviation.

**Table 2:** Inhibitory potentials of fennel extracts on *Staphylococcus aureus* and *Escherichia coli.*

Fennel Extract	Positive Effect (×)		Concentration (mg DE/mL)	Inhibition Zone Diameter
	*S. aureus*	*E. coli*		(mm)
LF	-	-	38.7	-
LF*	-	-	160	-
SF	×	-	110	10.00 ± 0.21
PF	×	-	20	10.50 ± 0.52

LF, liquid fraction used directly; LF*, liquid fraction at 40 °C; SF, solid fraction extract; PF, pellet fraction extract. (-) no inhibition zone was observed; (×), positive effect was observed.

**Table 3:** Antimicrobial activity of fennel solid fraction extract, SF (concentrations: 50 mg DE/mL = 5%, 100 mg DE/mL = 10%, and 150 mg DE/mL = 15%) and pellet fraction extract, PF (concentrations: 10 mg DE/mL = 1% and 20 mg DE/mL = 2%) on *Salmonella enterica* serotype Enteritidis*, Escherichia coli*, *Pseudomonas aeruginosa*, *Staphylococcus aureus*, and *Bacillus cereus* growth and comparison with the positive control (PC).

Indicator Strains	SF	PF
*Salmonella enterica* serotype Enteritidis	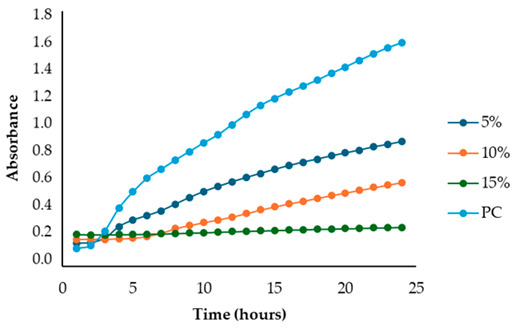	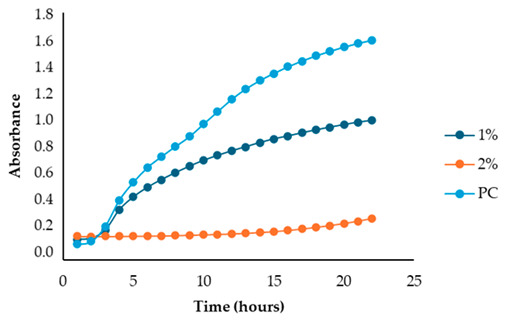
*Escherichia coli*	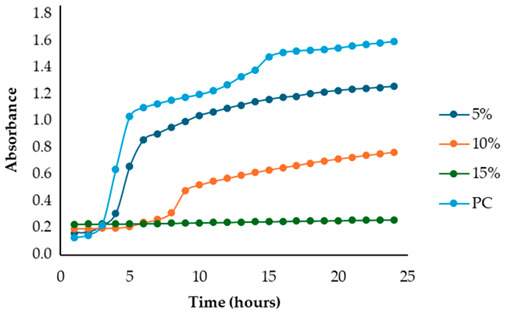	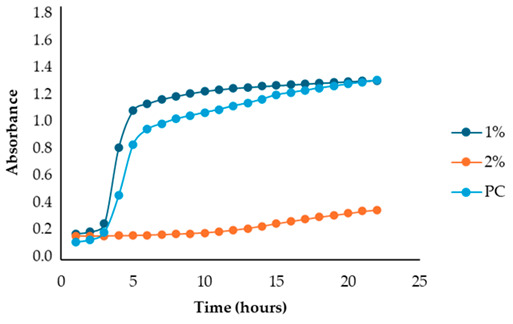
*Pseudomonas aeruginosa*	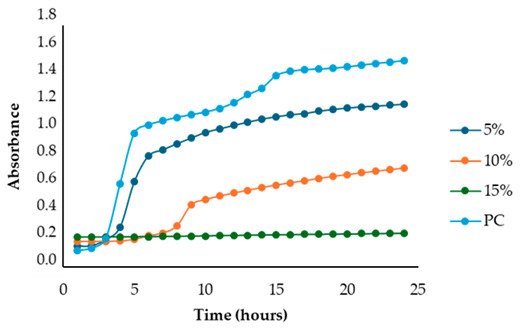	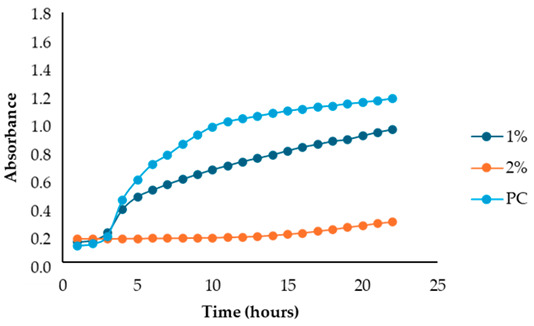
*Staphylococcus aureus*	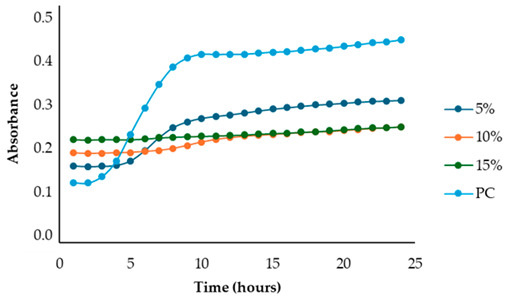	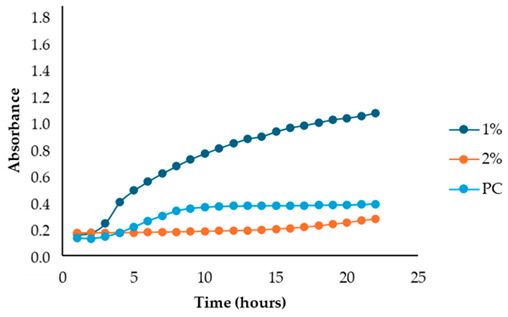
*Bacillus cereus*	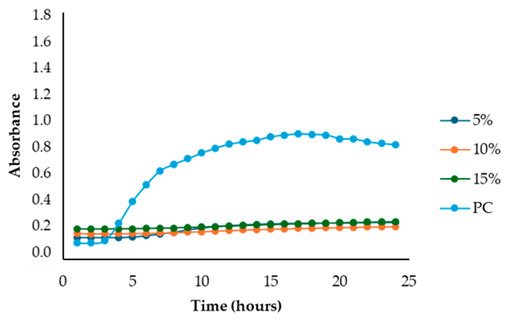	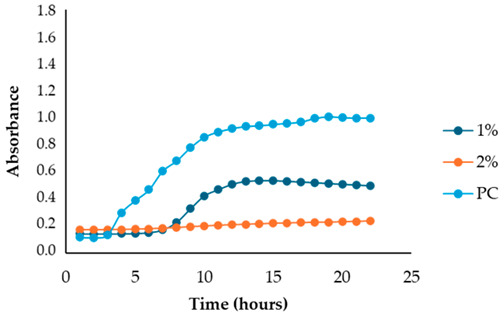

**Table 4:** Inhibitory potentials of the solid fennel (SF) and pellet extract (PF) on *Salmonella enterica* serotype Enteridis.

Item	SF			PF	
	50 mg DE/mL (5%)	100 mg DE/mL (10%)	150 mg DE/mL (15%)	10 mg DE/mL (1%)	20 mg DE/mL (2%)
Absorbance	0.843	0.556	0.271	1.013	0.300
Inhibition index, %	43.82	62.92	81.92	36.57	81.21

The absorbance value and the calculation of inhibition index (%) were recorded after 22 h.

**Table 5:** Inhibitory potentials of the solid fennel (SF) and pellet extract (PF) on *Escherichia coli.*

Item	SF			PF	
	50 mg DE/mL (5%)	100 mg DE/mL (10%)	150 mg DE/mL (15%)	10 mg DE/mL (1%)	20 mg DE/mL (2%)
Absorbance	1.199	0.717	0.253	1.013	0.300
Inhibition index, %	21.01	52.74	83.31	0.117	72.189

The absorbance value and the calculation of inhibition index (%) were recorded after 22 h.

**Table 6:** Inhibitory potentials of the solid fennel (SF) and pellet extract (PF) on *Pseudomonas aeruginosa*.

Item	SF			PF	
	50 mg DE/mL (5%)	100 mg DE/mL (10%)	150 mg DE/mL (15%)	10 mg DE/mL (1%)	20 mg DE/mL (2%)
Absorbance	0.820	0.475	0.253	0.956	0.291
Inhibition index, %	33.44	61.42	79.44	18.91	75.27

The absorbance value and the calculation of inhibition index (%) were recorded after 22 h.

**Table 7:** Inhibitory potentials of the solid fennel (SF) and pellet extract (PF) on *Staphylococcus aureus*.

Item	SF			PF	
	50 mg DE/mL (5%)	100 mg DE/mL (10%)	150 mg DE/mL (15%)	10 mg DE/mL (1%)	20 mg DE/mL (2%)
Absorbance	0.298	0.240	0.242	1.027	0.266
Inhibition index, %	29.30	42.90	42.58	-	28.30

The absorbance value and the calculation of inhibition index (%) were recorded after 22 h.

**Table 8:** Inhibitory potentials of the solid fennel (SF) and pellet extract (PF) on *Bacillus cereus*.

Item	SF			PF	
	50 mg DE/mL (5%)	100 mg DE/mL (10%)	150 mg DE/mL (15%)	10 mg DE/mL (1%)	20 mg DE/mL (2%)
Absorbance	0.266	0.236	0.274	0.498	0.236
Inhibition index, %	68.92	72.42	67.99	50.17	76.36

The absorbance value and the calculation of inhibition index (%) were recorded after 22 h.

**Table 9:** Microbiological ((Log (CFU/g)) results of treated (TRT) and untreated (CTR) beef burgers.

Item		0 d	5 d	8 d	12 d	15 d	18 d	Limits	RE
TAB 30 °C	TRT	2.36	2.80	3.26	3.71	5.32	5.32	m = 5.69	Reg. CE 2073/05 s.m.i. 1441/07
	CTR	2.43	2.84	3.34	3.81	5.50	5.52	M = 6.69	
Total Coliforms	TRT	<1.0	<1.0	<1.0	<1.0	<1.0	<1.0		
	CTR	<1.0	<1.0	<1.0	<1.0	<1.0	<1.0		
β-glucuronidase-positive *Escherichia coli*	TRT	<1.0	<1.0	<1.0	<1.0	<1.0	<1.0	m = 2.69	Reg. CE 2073/05 s.m.i. 1441/07
	CTR	<1.0	<1.0	<1.0	<1.0	<1.0	<1.0	M = 3.69	
Yeasts and molds	TRT	<1.0	<1.0	<1.0	<1.0	1.56	1.56		
	CTR	<1.0	<1.0	1.1	1.32	1.61	1.63		
Coagulase-positive Staphylococci	TRT	<1.0	<1.0	<1.0	<1.0	<1.0	<1.0		
	CTR	<1.0	<1.0	<1.0	<1.0	<1.0	<1.0		
*Listeria monocytogenes*	TRT	<1.0	<1.0	<1.0	<1.0	<1.0	<1.0	2.00	Reg. CE 2073/05 s.m.i. 1441/07
	CTR	<1.0	<1.0	<1.0	<1.0	<1.0	<1.0		
pH	TRT	5.81 ± 0.02	5.70 ± 0.03	5.59 ± 0.02	5.48 ± 0.01	5.45 ± 0.01	5.40 ± 0.03		
	CTR	5.81 ± 0.02	5.73 ± 0.03	5.65 ± 0.02	5.53 ± 0.01	5.45 ± 0.02	5.37 ± 0.02		
a_w_	TRT	0.979 ± 0.000	0.980 ± 0.002	0.981 ± 0.001	0.975 ± 0.006	0.976 ± 0.006	0.975 ± 0.004		
	CTR	0.979 ± 0.000	0.979 ± 0.001	0.980 ± 0.002	0.980 ± 0.000	0.975 ± 0.009	0.974 ± 0.003		

TRT, treated beef burgers during cold storage; CTR, untreated beef burgers during cold storage; TAB 30 °C, total aerobic plate count 30 °C.

## Data Availability

The data presented in this study are available upon request from the corresponding authors.
